# Risk behaviour associated with contracting the Ebola virus at funerals: a study of Liberians attending the funerals of individuals deceased from Ebola

**DOI:** 10.3389/fpubh.2026.1735066

**Published:** 2026-03-06

**Authors:** Moses Tende Stephens, Arunrat Tangmunkongvorakul, Natthapol Kosashunhanan, Mosoka Fallah, Julius S. M. Gilayeneh, Timothy Kie, Kriengkrai Srithanaviboonchai

**Affiliations:** 1 Research Institute for Health Sciences, Chiang Mai University, Chiang Mai, Thailand; 2 Center for Disease Control and Prevention (Africa CDC), Addis Ababa, Ethiopia; 3 National Public Health Institute of Liberia, Monrovia, Liberia; 4 African Methodist Episcopal University, Monrovia, Liberia; 5 Faculty of Medicine, Chiang Mai University, Chiang Mai, Thailand

**Keywords:** Ebola virus disease, funeral practices, infectious disease transmission, Liberia, risk behaviour

## Abstract

**Introduction:**

Funeral practices were identified as a significant source of Ebola transmission during the 2014–2016 West Africa Ebola outbreak. This study investigated behaviours and contributing factors leading to Ebola exposure during funerals in Montserrado County, Liberia.

**Methods:**

In 2024, a cross-sectional study was conducted using face-to-face interviews with 200 participants (mean age:51 years; 69.5% male) from Clara Town and Vai Town with family members of EVD victims and community leaders in Clara Town and Vai Town.

**Results:**

High-risk behaviour, defined as kissing or touching the deceased without gloves, was reported by 29.5% of participants. In unadjusted analysis, males (COR = 2.40, 95% CI: 1.14–5.03), the Kpelle tribe (COR = 3.17, 95% CI: 1.66–6.05), Christians (COR = 2.69, 95% CI: 1.42–5.09), and community leaders (COR = 2.31, 95% CI: 1.23–4.32) were more likely to engage in high-risk behaviours. In multivariate analysis, only religion remained independently associated; Christians were more likely than Muslims to engage in such behaviours (AOR = 2.70, 95% CI: 1.40–5.18).

**Discussion:**

Kissing the deceased was the main risk behaviour. The study highlights the influence of cultural traditions on Ebola spread and calls for culturally sensitive prevention strategies.

## Introduction

The Ebola virus is an RNA virus with one strand categorised within the Ebolavirus genus (1). Four known species are deemed harmful to humans: Zaire, Sudan, Taï Forest, and Bundibugyo (2). Direct contact with the bodily fluids of sick people, including blood, saliva, vomit, urine, and faeces, allows Ebola to be spread. Furthermore, contaminated surfaces and objects, such as medical tools and clothes, represent possible means of transmission (3).

Ebola Virus Disease (EVD) has an incubation period from 2 to 21 days (4, 5), and a case fatality rate as high as 90% (6). The pathophysiology of EVD involves vascular inflammation and damage induced by cytokine release (7). The disease is characterised by fever, gastrointestinal and respiratory disturbances, rash, and hemorrhagic manifestations (8).

The 2014–2016 West African EVD epidemic, the most widespread outbreak to date, had a profound impact on Liberia, Guinea, and Sierra Leone (9). Despite the spread of cases to other nations, the majority of infections and fatalities were concentrated in these three countries, with over 28,600 cases and 11,000 deaths (10, 11). The lower-than 90% case fatality rates during this epidemic reflects differences in viral strain, timing of case detection, access to supportive care, availability of treatment facilities, and community-level public health interventions.

The outbreak was exacerbated by insufficient knowledge regarding EVD among the population and inadequate resources and infrastructure to manage the pandemic (12). The scarcity of essential protective equipment, such as gloves, gowns, and face shields, as well as the insufficient training of health staff, worsened the challenges faced during the outbreak (13).

Liberia was especially hard hit during the outbreak, with 10,675 confirmed cases and 4,810 reported deaths (14). Montserrado County, Liberia’s most populous and economically vital region, and the location of the capital city, Monrovia, was the area most severely affected by the epidemic, resulting in numerous fatalities and substantial repercussions for the local population (15). The EVD epidemic in Montserrado County highlights the interplay of public health, knowledge, and cultural practices (16).

Funeral practices have been identified as key transmission routes for EVD (17). Traditional funeral practices in Africa often involve close physical contact with the deceased, including bathing, dressing, and even kissing the body (18). Blevins et al. (19) found that the transmission was driven by religious and traditional burial practices, including washing and wrapping bodies. These practices pose significant risks for Ebola transmission, as the virus remains highly infectious in bodily fluids even after death. One specific funeral in Sierra Leone was identified as an essential source of Ebola transmission, resulting in multiple infections and deaths (20).

In Liberia, funerals are deeply spiritual events that often involve contact with the deceased, which can conflict with public health prevention measures (21). Among the Kpelle and Mandingo communities, two of the primary ethnic groups in Montserrado County, funeral practices hold profound cultural meaning (22). Traditional funeral rites among these groups, including bathing, dressing, and kissing the body, significantly increased the risk of EVD transmission (23).

Despite the WHO’s efforts to encourage safe burial practices, deeply rooted cultural traditions posed significant barriers to behavioural change (24). Community attitudes influenced intervention success; some accepted safe burials, while others saw them as disrespectful or externally imposed (25). Public health measures, including safe burial protocols, often met resistance due to cultural sensitivities (17), reflecting a broader tension between traditional norms and modern health interventions (26). The situation was further complicated by widespread misinformation, with some communities believing EVD was fabricated for political or economic purposes, which led to practices such as secret burials that undermined epidemic control efforts (27). Efforts to modify these deeply ingrained customs posed significant challenges to public health responses (28).

Although unsafe funeral practices have been identified as risk behaviours for Ebola transmission in the literature, they remain under-researched. This study aimed to examine these high-risk behaviours and the characteristics of individuals involved in them during the funerals of Ebola victims. The focus was on participants from Clara Town (predominantly Mandingo communities) and Vai Town (mainly Kpelle communities) in Montserrado County, who took part in such funerals during the 2014–2016 Ebola epidemic.

## Methods and materials

### Study design

This study utilises a cross-sectional quantitative approach.

### Study setting

The research was conducted in Clara Town and Vai Town, Montserrado County, Liberia, from September to December 2024. Liberia continues to face structural public health challenges following the 2014–2016 Ebola epidemic, including limited health infrastructure, constrained surveillance capacity, and overlapping infectious disease threats. Clara Town and Vai Town are densely populated urban communities in Montserrado County, where poverty, overcrowding, and reliance on traditional social structures influence health-related behaviours, including funeral practices.

### Participants

The majority of Clara Town’s residents are Mandingo, whereas the majority of Vai Town residents are Kpelle. The study’s target participants included community leaders (local zonal heads, youth groups, health facility representatives, church-based organisations, and community task force groups) as well as relatives of patients who died of the Ebola Virus Disease. Those two groups of people who attended the funerals of EVD deaths between 2014 and 2016, who were in Vai or Clara Town, were at least 30 years old, and of the Mandingo or Kpelle ethnicities, were eligible to participate in the study. The age restriction was set at 30 years or older because those younger than that were culturally expected not to engage in funeral and burial customs during the 2014–2016 EVD outbreak.

### Sampling strategy and sample size

To estimate the required sample size, 200 participants were selected, assuming a 50% prevalence of high-risk funeral behaviour and a 10% margin of error. However, participant recruitment was ultimately guided by feasibility and accessibility, given the study’s retrospective nature and the difficulty of identifying eligible individuals nearly 10 years after the outbreak. As such, the study employed a non-probability sampling approach. Participants were recruited through snowball sampling, which was appropriate for locating individuals who participated in Ebola-related funerals almost a decade earlier. Initial participants were selected from diverse social roles, including community leaders and family members, to reduce clustering and improve variation within the sample. The investigator encouraged the initial participants to refer others they were aware of who had taken part in funerals during the Ebola epidemic.

Snowball sampling was employed in this study due to the sensitive and retrospective nature of the topic, as well as the challenge of locating individuals who had participated in Ebola-related funerals nearly 10 years after the outbreak. To reduce potential bias, we selected initial participants from various social positions, including community leaders and ordinary family members, in the two study communities. Recruitment was monitored to ensure a balanced sample, with 100 participants enrolled from each site. This strategy helped assure diversity within the sample while maintaining methodological feasibility given the study’s context.

### Measurements

Demographic information collected included gender (female or male), age (year), tribe (Town), religion (Muslim, Christian, no religion), marital status at the time of the survey (single, married, widowed, separated/divorced), level of education (elementary school, high school, bachelor’s degree or higher), and type (community leader or family member). The primary outcome of the study is high-risk behaviour associated with funerals. High-risk behaviour was operationalised as either kissing the corpse or touching without gloves, following WHO and CDC outbreak guidance that identifies these acts as high-risk contact. The high-risk behaviour of “touching the deceased without gloves” was determined based on participants who answered “yes” to the first of the following questions and “no” to the second: (1) “Have you touched the dead body of an individual who died from Ebola virus disease during a funeral?” and (2) “Have you touched the dead body of an individual who died from Ebola virus disease without glove a funeral?.” To enhance content validity, the questionnaire underwent pilot testing with 12 individuals in Bomi County, a demographically similar region not included in the study sample. Minor modifications were made based on feedback to improve clarity and cultural appropriateness.

### Data collection

The lead investigator and a trained research assistant, both Liberian, conducted in-person interviews with participants. Responses were recorded and compiled using REDCap (Research Electronic Data Capture). A structured questionnaire was used to collect data from participants. The questionnaire was developed specifically for this study, based on the research objectives and relevant literature, and has not been previously published.

### Statistical analysis

The data were initially examined using SPSS version 22.0, a software program created by IBM in Armonk, NY, USA. Percentages, means, and frequencies were used to summarise the results. Differences in demographic characteristics between participants from Clara Town and Vai Town were assessed using Pearson’s chi-square test. The univariate analysis examined all demographic variables associated with high-risk behaviours and presented as Crude Odds Ratios (COR). Variables that showed significant associations with the primary outcome in univariate analysis were included in the multiple logistic regression model, following checks for multicollinearity. Adjusted Odds Ratios (AOR) were used to identify independent predictors of high-risk behaviour during funerals. This variable selection method was used to reduce model complexity and avoid overfitting, considering the modest sample size (*N* = 200). Gender, religion, ethnicity, and participant role were initially assessed; only those with significant associations were retained in the final model. For both the univariate and multivariate analyses, high-risk behaviour was used as dependent variable, 95% confidence intervals were calculated, and a *p*-value of less than 0.05 was considered statistically significant.

### Ethical considerations

Both national and institutional standards guided the ethics review process. Ethical approval was obtained from the Liberia National Ethics Committee (FWA00032198) and Chiang Mai University’s Faculty of Medicine Research Ethics Committee (COM-2567-0323). Written informed consent was obtained from all participants, and interviews were conducted in adherence to locally appropriate consent and confidentiality practices. All participants were above the age of 18.

## Results

Table 1 presents the demographic characteristics of participants stratified by community. Significant differences were observed between Clara Town and Vai Town with respect to gender and religion. Vai Town had a higher proportion of male participants (77.0%) than Clara Town (62.0%; *p* = 0.041). Religious affiliation differed markedly between communities: Clara Town was predominantly Muslim (71.0%), while Vai Town was predominantly Christian (78.0%) (*p* < 0.001). No statistically significant differences were observed between the two communities for age distribution, marital status, educational level, or participant type.

**Table 1:** Basic demographic characteristics of participants by community (*N* = 200).

Characteristics	Total *n* (%)	Clara Town (Mandingo) *n* (%)	Vai Town (Kpelle) *n* (%)	*p*-value
Gender				0.041
Female	61 (30.5)	38 (38.0)	23 (23.0)	
Male	139 (69.5)	62 (62.0)	77 (77.0)	
Age group (years)				0.318
30–39	31 (15.5)	17 (17.0)	14 (14.0)	
40–49	57 (28.5)	30 (30.0)	27 (27.0)	
50–59	64 (32.0)	29 (29.0)	35 (35.0)	
≥60	48 (24.0)	24 (24.0)	24 (24.0)	
Religion				<0.001
Muslim	89 (44.5)	71 (71.0)	18 (18.0)	
Christian	104 (52.0)	26 (26.0)	78 (78.0)	
No religion	7 (3.5)	3 (3.0)	4 (4.0)	
Marital status				0.214
Single	69 (34.5)	33 (33.0)	36 (36.0)	
Married	81 (40.5)	44 (44.0)	37 (37.0)	
Widowed	10 (5.0)	4 (4.0)	6 (6.0)	
Separated/Divorced	40 (20.0)	19 (19.0)	21 (21.0)	
Education level				0.089
Elementary school	9 (4.5)	6 (6.0)	3 (3.0)	
High school	96 (48.0)	44 (44.0)	52 (52.0)	
Bachelor’s degree or higher	95 (47.5)	50 (50.0)	45 (45.0)	
Type of participant				1.000
Family member	100 (50.0)	50 (50.0)	50 (50.0)	
Community leader	100 (50.0)	50 (50.0)	50 (50.0)	

Table 2 presents funeral-related risk behaviours stratified by community. Overall, 39.5% of participants reported engaging in at least one high-risk funeral behaviour. High-risk practices were more frequently reported in Clara Town than in Vai Town (49.0% vs. 30.0%, *p* = 0.006). Kissing the corpse was reported by 29.0% of participants, while 17.0% reported touching the corpse without gloves. Touching the body with gloves was common in both communities, reflecting partial adherence to infection prevention guidance. Body washing was reported by over one-third of participants, with a higher prevalence in Clara Town, although this difference did not reach statistical significance.

**Table 2:** Funeral-related risk behaviours by type and community (*N* = 200).

Risk behaviour	Total *n* (%)	Clara Town *n* (%)	Vai Town *n* (%)	*p*-value
Touched corpse without gloves (high-risk)	34 (17.0)	22 (22.0)	12 (12.0)	0.048
Kissed corpse (high-risk)	58 (29.0)	36 (36.0)	22 (22.0)	0.031
Touched the corpse with gloves	112 (56.0)	51 (51.0)	61 (61.0)	0.147
Participated in body washing	76 (38.0)	44 (44.0)	32 (32.0)	0.079
Any high-risk behaviour^a^	79 (39.5)	49 (49.0)	30 (30.0)	0.006

a High-risk behaviour is defined as kissing the corpse and/or touching the body without gloves.

*p*-values calculated using Pearson’s chi-square *t*.

**Figure 1:**
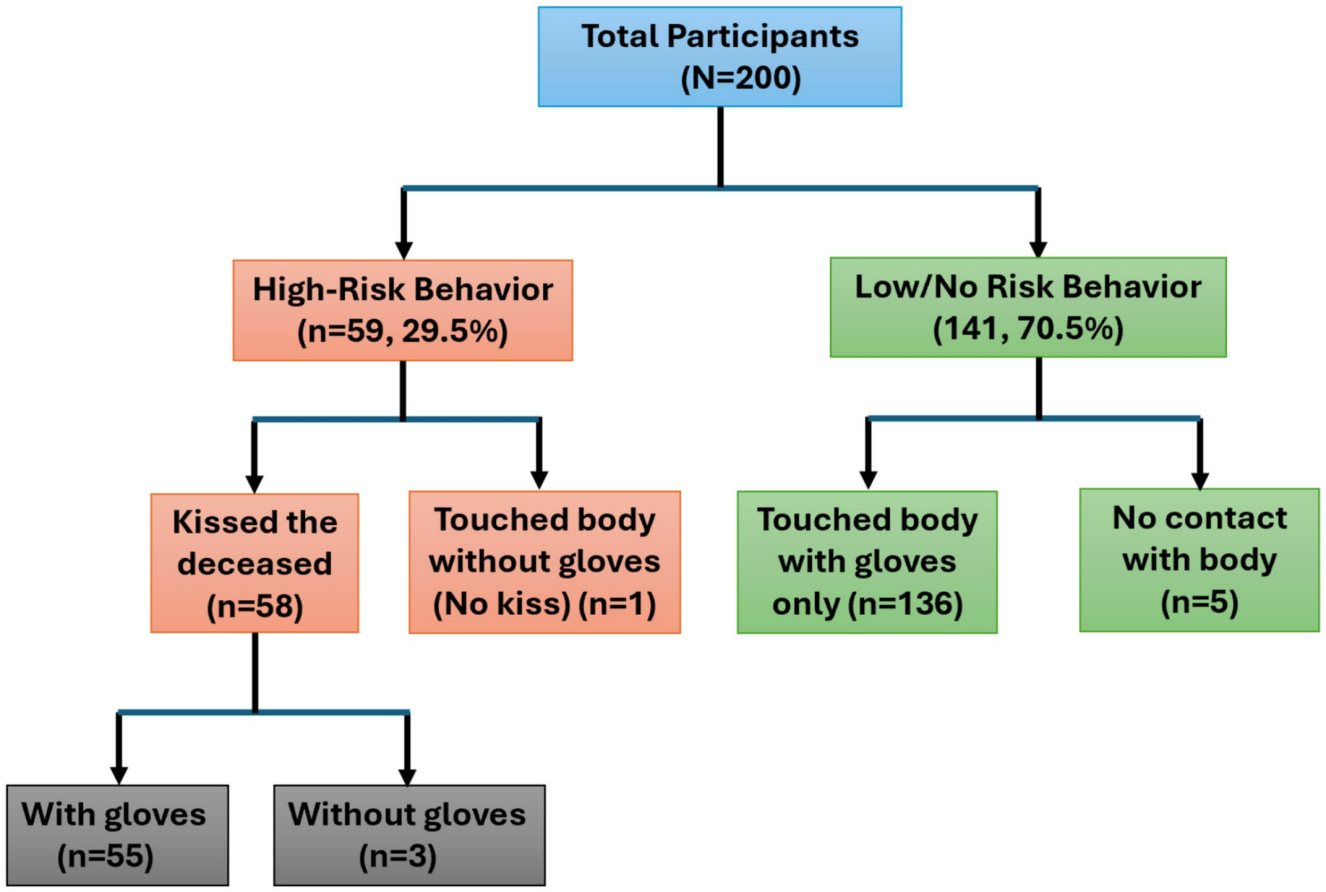
Risk behaviours of contracting Ebola during funerals.

Of the 200 participants, 195 (97.5%) reported physical contact with the deceased. Among those who reported contact, 191 individuals utilized gloves, while 4 individuals touched the body without gloves. Additionally, 58 participants reported kissing the deceased. High-risk behaviour, defined as either kissing the deceased or touching the body without gloves, was identified in 59 participants (29.5%). Within this high-risk group, 58 individuals reported kissing the deceased, a group that included 3 of the 4 individuals who also touched the body without gloves. The remaining high-risk participant was a single individual who touched the body without gloves but did not engage in kissing. Conversely, 136 participants limited their interaction to touching the body with gloves only, and 5 participants reported no physical contact with the deceased (Figure 1).

Analyses were conducted to examine the characteristics associated with high-risk behaviours related to funeral practices and EVD. The findings reveal that males exhibited significantly higher risk behaviours in the unadjusted analysis than females (COR = 2.40, 95% CI: 1.14–5.03). Participants from Kpelle (Via town) demonstrated significantly higher risk behaviours compared to those from Mandingo (Clara town) (COR = 3.17, 95% CI: 1.66–6.05). Christians exhibited significantly higher risk behaviours than Muslims (COR = 2.69, 95% CI: 1.42–5.09). Community leaders showed substantially higher risk behaviours than family members (COR = 2.31, 95% CI: 1.23–4.32). After adjusting for potential confounding using multivariate analysis, religion was the only factor independently associated with high-risk behaviour. Christians exhibited significantly higher risk behaviours than Muslims (AOR = 2.70, 95% CI: 1.40–5.18) (Table 3).

**Table 3:** Factors associated with high-risk behaviour related to funeral practice and Ebola virus disease (*N* = 200).

Characteristics	*n*/*N* (%)	COR (95% CI)	AOR (95% CI)
Gender			
Female	11/61 (18.0)	1	1
Male	48/139 (34.5)	2.40 (1.14–5.03)^a^	1.74 (0.76–4.02)
Age (years)			
30–39	8/31 (25.8)	1	^b^
40–49	12/57 (21.1)	0.77 (0.28–2.14)	
50–59	19/64 (29.7)	1.21 (0.46–3.19)	
≥60	20/48 (41.7)	2.05 (0.76–5.52)	
Tribe (Region)			
Mandingo (Clara town)	18/100 (18.0)	1	^b^
Kpelle (Vai town)	41/100 (41.0)	3.17 (1.66–6.05)^a^	
Religion			
Muslim	21/104 (20.2)	1	1
No religion	2/7 (28.6)	1.58 (0.29–8.73)	2.20 (0.38–12.83)
Christian	36/89 (40.4)	2.69 (1.42–5.09)^a^	2.70 (1.40–5.18)*
Marital status			
Single	19/69 (27.5)	1	^b^
Married	29/81 (35.8)	1.47 (0.73–2.95)	
Widowed	3/10 (30.0)	1.13 (0.26–4.82)	
Separated/divorced	8/40 (20.0)	0.66 (0.26–1.68)	
Level of education			
Elementary school	4/9 (44.4)	1.73 (0.43–6.92)	^b^
High school	25/96 (26.0)	0.76 (0.41–1.43)	
Bachelor’s degree or Higher	30/95 (31.6)	1	
Type of participant			
Family member	21/100 (21.0)	1	1
Community leader	38/100 (38.0)	2.31 (1.23–4.32)^a^	1.91 (0.93–3.93)

a Statistically significant.

b Not included in the multivariate analysis.

## Discussion

Risky behaviours during the funerals of individuals who died from EVD have been well-established as a significant route of Ebola transmission in Africa. This is the first study to identify and document the factors associated with these practices. The study’s findings revealed that almost all of the participants (97.5%) had direct contact with the bodies of individuals who had died from Ebola during the funeral process. However, 97.9% of these participants reported utilising precautionary measures, such as wearing gloves during those acts, and were not deemed to have engaged in risky behaviour. Finally, approximately 30% of participants engaged in high-risk activities, with kissing identified as the most significant behaviour. Nearly all individuals in this group (58 out of 59) reported kissing the corpses. Kissing during funerals, though a culturally significant act of farewell, poses a high risk for Ebola Virus Disease (EVD) transmission due to direct contact with the infectious body. Most reports, including those by WHO, broadly mention “close contact” without isolating kissing. This study is among the first to report kissing as a specific EVD risk behaviour. While glove use likely reduced direct exposure, it may also have encouraged prolonged or closer contact with the body by creating a false sense of safety, particularly when gloves were not used or removed correctly.

In this present study, religion has been identified as an independent predictor of engaging in risky behaviours during the funerals of EVD victims. Christians demonstrated significantly higher levels of risky funeral practices compared to Muslims. Existing literature links direct contact during funerals to EVD transmission, but it is rarely distinguished by religion. While the link between funeral practices and Ebola Virus Disease (EVD) transmission is well known, this study introduces several novel empirical and theoretical contributions. It is among the first to statistically demonstrate that Christian funeral rites, particularly the act of kissing the deceased, are independently associated with high-risk behaviours compared to Muslim practices, offering new insights for culturally and faith-sensitive interventions. Unlike most prior research that broadly categorises “close contact,” this study identifies “kissing the corpse” as a specific and culturally significant act, accounting for 98% of reported high-risk behaviours. A substantial proportion of community leaders were male (90%), reflecting local leadership structures. This overlap suggests that gender and leadership roles may be interrelated in shaping exposure to funeral practices.

In Liberia, funerary practices vary by religious affiliation, with important implications for Ebola virus disease (EVD) transmission risk. Muslim rites typically emphasise ritual washing, shrouding, and prayer, and generally do not include kissing the deceased. In contrast, Christian funeral services more often involve viewing and closer physical interaction, including touching or kissing the body as a sign of respect and farewell (29, 30). Kissing is not a doctrinal requirement in Christian practice, and abstaining from physical contact is generally acceptable to families and communities when justified by health concerns (31). During the Ebola outbreak, the continuation of such practices appeared to reflect limited awareness of transmission risks rather than strict religious obligation (32). Risk reduction efforts should therefore focus on modifiable behaviours, particularly kissing and direct contact with the body, while preserving core religious values. Feasible, faith-congruent alternatives include verbal blessings and prayers performed at a distance; symbolic gestures such as placing a cloth or flowers near the deceased; supervised body preparation by trained personnel using personal protective equipment; and, where necessary, temporary substitutions for ritual washing, such as tayammum or symbolic wiping over the shroud (33). Collaboration with clergy, faith leaders, older adults in the community, and burial team supervisors is essential to communicate the religious acceptability of these temporary adaptations and to support dignified and protective mourning practices during epidemic outbreaks.

The higher engagement in risky funeral practices among the Kpelle ethnic group may also be influenced by religious affiliation, as most Kpelle are Christian. This aligns with our findings that Christian funeral rituals, such as touching or kissing the deceased, are associated with increased EVD transmission risk. In the Kpelle community, particularly in Vai Town, there is a firm adherence to cultural traditions that involve direct physical contact with the deceased. These practices are essential to the community’s expressions of mourning and respect for the dead. The increased risk behaviours among the Kpelle emphasise the urgent need for culturally sensitive public health interventions that respect traditional values while minimising risk. Previous studies (34) have highlighted the impact of ethnic-specific practices on exposure to infections during funerals, suggesting that public health guidelines should be adapted to fit local cultural contexts to improve adherence and effectiveness.

Although the adjusted analysis showed the association was no longer significant, the crude analysis indicated that males were more likely than females to engage in high-risk funeral behaviours. This trend suggests that cultural norms and social expectations among men may predispose them to engage in traditional funeral practices involving direct contact with the deceased. Men in these communities have been more involved in ritualistic practices that necessitate handling the body, a role potentially derived from social responsibilities assigned during mourning ceremonies (35).

Community leaders, when compared to family members, were found to engage more frequently in high-risk behaviours during funerals in crude analysis. This finding can be interpreted in several ways. Community leaders often have a dual role: preserving cultural heritage and serving as role models. Their participation in traditional practices could reinforce risky behaviours among the broader population, but they also represent a strategic leverage point for intervention. Previous studies (36) suggested that involving community and religious leaders in public health campaigns can significantly enhance compliance with safe practices.

Our findings imply that interventions to reduce the risk of Ebola transmission during funerals should be culturally informed and community-driven for public health practice (19). This emphasised the complex interplay between cultural beliefs and public health initiatives. As kissing the deceased was identified as a high-risk practice, this study highlights the urgent need for culturally sensitive strategies to reduce such behaviour. Interventions should engage religious leaders, older adults in the community, and community health workers to promote safer alternatives that preserve the intent of farewell rituals. Trusted local figures can guide action, while the health education guided by the Health Belief Model (37) can emphasise perceived susceptibility and severity. Utilising the Health Belief Model, we can elucidate the participants’ choices regarding high-risk funeral practices, especially touching or kissing the deceased, as follows. Perceived susceptibility was often low, as many individuals believed they were spiritually protected or underestimated their personal risk of contracting Ebola. While some participants acknowledged the severity of the disease, they still prioritised fulfilling cultural and religious rites. Perceived barriers such as strong cultural expectations, spiritual obligations, and fear of social exclusion limited behaviour change. Cues to action, including public health messages from religious leaders and government mandates during the outbreak, played a key role in shaping compliance with safe burial practices. Low perceived susceptibility may also be explained by the belief that a deceased person poses less risk than a visibly ill individual. This misconception highlights the need for clear, funeral-specific public health guidance during future outbreaks.

Touching, washing, or kissing the corpse carries deep symbolic meaning in many cultures, representing respect, farewell, and spiritual continuity. As noted by (33), Corpses’ rituals often serve social and emotional functions that reinforce communal bonds, making behavioural change during epidemics particularly challenging.

Empowering communities through respectful dialogue is essential for modifying risky traditions without cultural alienation. Public health initiatives can gain greater traction in communities where traditional and modern health practices intersect by focusing on factors such as perceived susceptibility and severity, as well as cues to action from trusted local figures. Christian funerals in Liberia typically include a blessing from a family leader. These individuals can be educated or trained to incorporate a health message toward the end of the blessing, emphasizing that touching and kissing the corpse is dangerous. They can also convey that abstaining from touching and kissing the corpse is spiritually acceptable as a new traditional guideline. During future epidemics, additional practices can be implemented. This may include the use of a physical barrier, such as placing a cloth around or near the deceased.

This study is particularly relevant for several reasons. Cultural appropriateness clarifies funeral customs and their role in the worsening of the Ebola pandemic. Focusing on multiple ethnic communities, specifically the Kpelle and Mandingo, enhances the comprehensiveness and localisation of the data. Collecting real-life data through face-to-face interviews allowed interviewers to provide clarifications, thereby improving the reliability of the information compared to self-administered questionnaires. A high response rate of 95% ensures comprehensive, unbiased data. Adherence to social norms and recognition of the study’s public health significance strengthen its ability to inform future initiatives and improve epidemic response. Although most participants reported wearing gloves when touching the body, kissing the deceased remained common. This suggests that glove use, which was widely promoted during the outbreak, may have reduced perceived risk while not fully addressing culturally meaningful practices such as kissing, which were not consistently recognised as hazardous.

### Practical implications

These results have concrete implications for Ebola preparedness and response. Identifying kissing the deceased as the most common high-risk practice enables more precise risk communication and community education, shifting from broad cautions about close contact to guidance that targets specific behaviours. Differences between Christian and Muslim funeral rituals further indicate the need for prevention strategies tailored to religious and cultural contexts. In Liberian Christian funerals, where a family leader often offers a final blessing, these individuals could be engaged and trained to weave brief health messages that explain the risks of touching or kissing the body during outbreaks and affirm that temporarily abstaining is spiritually acceptable. Faith leaders, older adults in the community, health workers, and burial team supervisors can also promote safe symbolic substitutes, such as spoken blessings, prayers, or the placement of a cloth near the deceased. Together, these measures can support community-led preparedness for future outbreaks and the design of evidence-based, culturally congruent, and operationally feasible interventions in outbreak settings. The findings have relevance beyond EVD, particularly for other highly infectious conditions involving corpse handling, such as Marburg virus disease and other emerging epidemic threats. Understanding culturally embedded funeral behaviours is critical for designing effective, context-specific interventions across infectious disease outbreaks.

### Study limitations

This study was conducted in 2024, nearly a decade after the 2014–2016 Ebola outbreak, which introduces potential challenges related to recall bias and historical accuracy. Participants may have experienced difficulties in accurately recalling specific behaviours during funeral ceremonies, especially when memory has been shaped by subsequent exposure to health education or media narratives. The questionnaire did not include items asking whether participants contracted Ebola or survived an infection. This decision reflected ethical sensitivity and the limitations of long-term recall. To mitigate this, the study focused on highly emotionally salient and culturally symbolic risk behaviours, such as kissing the deceased, which are more likely to be retained in long-term memory. Literature suggests that emotionally significant or traumatic public health events are more vividly remembered and reconstructed with reasonable accuracy over time. The study did not assess whether gloves were used or removed correctly, which is an essential consideration because improper removal can still result in exposure. Additionally, social desirability bias may have influenced participants’ willingness to disclose risky behaviours now widely understood as unacceptable. To reduce this bias, interviews were conducted privately by trained Liberian researchers in nonjudgmental, culturally sensitive language. Participants were also assured of confidentiality and anonymity and informed that their responses would have no personal or legal consequences. While snowball sampling is efficient, it can introduce selection bias by attracting individuals who are more engaged in their communities or who share particular beliefs. Individuals who engaged in the highest-risk behaviours during the outbreak may have died and therefore could not be included in the study, potentially resulting in survivor bias and underestimation of risk behaviours. Gender norms within these communities may have influenced both participation and reporting, as men were more likely to be involved in funeral rituals and to engage with researchers, introducing possible participation bias.

Snowball sampling was used because of the sensitive and retrospective nature of the topic. To reduce homogeneity and clustering of similar practices, initial participants were purposively selected from diverse social roles, including community leaders and family members, across both study communities. Despite these efforts, the potential for selection bias remains.

## Conclusion

The study identified the practice of kissing the bodies of the deceased during funerals as a significant potential route of Ebola transmission within the communities. It also found that Christians were associated with higher risk behaviours during funeral practices. The findings highlight how cultural and religious traditions influenced behaviours that contributed to the spread of EVD. Future epidemic preparedness and response strategies in these areas should address kissing as a main risk behaviour and incorporate culturally sensitive approaches that respect local traditions while promoting effective disease prevention. Interventions aimed at preventing high-risk behaviour during funerals should be culturally sensitive and context-specific. Such interventions should actively engage religious leaders, older adults in the community, and health workers in co-developing and promoting safe symbolic alternatives to physical contact with the deceased, including verbal blessings, ritualized prayers, or the placement of symbolic items, such as a cloth, near the body.

## Data Availability

The raw data supporting the conclusions of this article will be made available by the authors, without undue reservation.
